# Financial Literacy and Gambling Behavior: Evidence from Japan

**DOI:** 10.1007/s10899-020-09936-3

**Published:** 2020-03-17

**Authors:** Somtip Watanapongvanich, Punjapol Binnagan, Pongpat Putthinun, Mostafa Saidur Rahim Khan, Yoshihiko Kadoya

**Affiliations:** Department of Economics, Hiroshima University, 1-2-1 Kagamiyama, Higashihiroshima, Hiroshima, 739-8525 Japan

**Keywords:** Gambling, Financial literacy, Financial education, Japan

## Abstract

According to a survey by Japan’s Ministry of Health, Labor, and Welfare in 2017, 3.6% of Japanese adults—equivalent to about 3.2 million people—have suffered from problem gambling at some point in their lifetime. This study examines the relationship between financial literacy, financial education, and gambling behavior (measured as gambling frequency) among the Japanese population. We hypothesize that financially literate and financially educated people who use their knowledge to make sound financial decisions are less likely to gamble. The data used in this study are from a nationwide survey in Japan from the Preference Parameters Study of Osaka University in 2010 (n = 3687). To control for endogeneity bias between financial literacy and gambling behavior, we use the education of respondents’ fathers as an instrumental variable. The results from the probit-instrumental variable model show that financial literacy has a significantly negative relationship with gambling frequency, while financial education has no significant relationship with gambling frequency. Our findings suggest that problem gambling may be mitigated by promoting financial literacy, but no such conclusion can be drawn for financial education.

## Introduction

Japan, the world’s third largest gambling market in 2016 with an overall per-country gambling loss of US $24.1 billion (Economist 2017), has been the subject of increasing concern following the country’s Integrated Resorts Implementation Bill, which became law in July 2018. The bill allows the establishment of casinos and gambling at integrated resorts to boost tourism and use the revenue earned to aid local economies. However, there is concern that the bill will lead to gambling-related problems (Asahi Shimbun 2018; Tanaka 2018). According to a survey by Japan’s Ministry of Health, Labor, and Welfare in 2017, 3.6% of Japanese adults—equivalent to about 3.2 million people—have suffered from problem gambling at some point in their life. Similar statistics for suspected problem gambling in other countries are much lower, in the range of 1–2%. Currently, 0.8% of the Japanese population exhibit behavior consistent with problem gambling—equivalent to around 700,000 people (Imai 2018; Osaki 2018; Tanaka 2018).

Gambling can have severely negative consequences (Calado and Griffiths 2016; Churchill and Farrell 2018). In terms of clinical implications, problem gambling is often associated with mental health disorders, such as depression, phobias, anti-social personality, alcoholism, and substance abuse and addiction. Another side effect of problem gambling is a marked increase in impulsive behavior (Cunningham-Williams et al. 1998; Haydock et al. 2015; Leeman and Potenza 2012; Moodie and Finnigan 2006). In addition, problem gambling can have other serious social and family costs, such as job loss, failed relationships, indebtedness, suicide, and crime (Cassetta et al. 2018; NAFGAH n.d.). Because of the growth in problem gambling, the number of individuals seeking assistance for gambling-related problems has increased exponentially. As a result, to minimize the effects of gambling-related issues, the need has increased for governments to allocate more of their budgets to intervention programs, such as those in health, welfare, employment, housing, and criminal justice (Calado and Griffiths 2016).

The rapid expansion of gambling has led to exponential growth in gambling-related research, with regard to both health and social consequences. The most prevalent topics related to gambling studies have been pathology, risk-taking, decision-making, and addiction (Eber and Shaffer 2000; Shaffer et al. 2006). In addition to these psychological and social issues, problem gambling has important economic consequences for the financial wellbeing of gamblers. Increased gambling losses place serious pressure on gamblers’ savings and investments. While gambling is associated with indebtedness (Alessi and Petry 2003) and impulsivity (Hurla et al. 2017; Leeman and Potenza 2012), financial literacy provides people with the ability to make informed decisions about financial planning, wealth accumulation, debt, and pensions. Higher levels of financial literacy enable people to be more financially secure in their retirement (Lusardi and Mitchell 2011, 2014). Financial literacy provides the opportunity for people to learn how to manage money, particularly through budgeting skills, understanding credit, and creating spending and savings plans (MCCG n.d.). Kadoya and Khan (2018) and Kadoya et al. (2018) provide evidence that financially literate people are able to make sound financial decisions about accumulating more assets and earning more income as a means of reducing anxiety about life in their old age. Thus, it is important to examine whether financial knowledge deters people from gambling. We hypothesize that financially literate people who use their knowledge to make sound financial decisions are less likely to gamble.

To the best of our knowledge, there is no empirical research addressing the association between financial literacy and gambling behavior. Our study contributes to the existing literature in at least two ways. First, we examine whether financial literacy reduces gambling frequency. Second, our research distinguishes between observed financial literacy and financial education received in school. This distinction allows us to understand whether economic values instilled in early school life have consequences on economic decisions made later in life. Our study has important policy implications as well. Our study adds to the understanding of the association between financial literacy, financial education, and gambling behavior; thus, we strongly consider that our findings can help policymakers in Japan devise effective means of intervention to prevent and minimize the negative consequences of gambling-related problems.

The rest of the paper is organized as follows. Section 2 reviews previous studies, while Sect. 3 discusses the data and methodology. Section 4 presents the empirical results, and Sect. 5 discusses these results. Section 6 concludes.

## Literature Review

### Gambling Behavior

The theory of addiction indicates that people are sometimes irrational because they choose to gamble, even though they can be much better off by not gambling (Herrnstein and Prelec 1992). Churchill and Farrell (2018) claim that people gamble because gambling is a fun-generating leisure activity, and a psychological factor accounting for utility enhancement has been added to the expected utility function. The point of switching from gambling to not gambling depends on the level of psychological effects that an individual experiences from gambling. If an individual’s utility associated with the psychological impacts of gambling is positive and outweighs the negative expected utility from gambling, the individual will continue to gamble (Churchill and Farrell 2018).

Hurla et al. (2017) adapted the theory of addiction from Herrnstein and Prelec (1992) to provide a behavioral model of gambling and categorized gamblers into three groups: recreational gamblers, frequent gamblers, and problem gamblers. In the first stage, a person gambles at a low rate and still enjoys other activities in life. In this case, gambling is under control and these gamblers are considered recreational gamblers. An equilibrium for recreational gamblers is when they have equal and relatively average enjoyment levels from both gambling and non-gambling activities (Herrnstein and Prelec 1992). The further an individual moves away from the equilibrium point, the less he or she enjoys gambling relative to non-gambling activities. However, some people choose to gamble because of other factors, such as stress relief and socializing with friends. As a result, a person may gamble more, and turns into a frequent gambler. Thereafter, if the person continues to gamble at a higher rate, they experience rapidly declining levels of enjoyment from both gambling and non-gambling activities as a result of financial problems, social disapproval, and consequences in their personal and professional lives. Finally, when they have a lower level of well-being from gambling, they become problem gamblers (Herrnstein and Prelec 1992).

Even though there are guidelines for *responsible gambling*, which is defined as a level of gambling that causes no harm, no participation limits are mentioned, such as gambling frequency and expenditure (Currie et al. 2006). Risk and harm from gambling are associated with the level of participation—the more time and money spent on gambling, the higher the risk of gambling-related harm (Calado and Griffiths 2016; Currie et al. 2006; Kessler et al. 2008; Manzenreiter 2013; Martin et al. 2010; Shaffer 2005).

Using the Canadian Community Health Survey and Canadian Problem Gambling Index (CPGI), Currie et al. (2006) estimated the low-risk limits of gambling, that is, where gamblers suffer minimal or no harm. The authors found that the low-risk limits of gambling based on participation level were gambling two to three times per month, spending 501–1000 CAD per year, and investing 1% of gross family income. If gamblers gamble more than the low-risk limits, there is a higher probability they will experience gambling-related harm. Moreover, Currie et al. (2006) found that risk level increased noticeably in gamblers who gamble more than once a week. For example, people who gamble more than the low-risk limit of two to three times per month, such as gambling at least once a week or more, are about 13 times more likely to experience gambling-related harm than individuals who gamble below the low-risk limit.

Gambling behavior is defined by aggregate gambling decisions over time (Hurla et al. 2017). Participation in gambling or gambling frequency is one of problem gambling’s risk factors (Calado and Griffiths 2016; Currie et al. 2006; Kessler et al. 2008; Manzenreiter 2013; Martin et al. 2010; Shaffer 2005). Hence, our study uses gambling frequency as a measurement of gambling behavior. We utilize the low-risk limits of gambling frequency that cause minimal or no harm for gamblers as proposed by Currie et al. (2006) to classify gamblers into groups following the behavioral model of gambling developed by Hurla et al. (2017). In our study, gamblers who gamble more than the low-risk limit of two to three times per month are defined as *“frequent gamblers”* since they face more negative consequences and enjoy gambling activities less. The details of the gambling behavior variable are discussed in the data section.

### Financial Literacy and Financial Education

Apart from the theory of addiction, hyperbolic discounting and time inconsistency are economic concepts that can be used to explain gambling behavior since people tend to value the present more than the future. In other words, individuals favor smaller, immediate rewards over larger, deferred rewards (Frederick et al. 2002; Hurla et al. 2017; Thaler 1981). This kind of economic behavior is also relevant when people make financial decisions because both gambling and financial decisions have an intertemporal nature. Moreover, in both circumstances, contextual influences affect gamblers or consumers with low expertise in making accurate risk assessments (Hurla et al. 2017).

The decision-making process for these issues is complex and requires financial knowledge; hence, financial literacy is key to helping people make informed decisions. According to Lusardi and Mitchell (2014, p. 6), financial literacy is “peoples’ ability to process economic information and make informed decisions about financial planning, wealth accumulation, debt, and pensions.” Existing studies show that financial literacy improves people’s ability to make decisions that are beneficial to their well-being (Braunstein and Welch 2002; Lusardi et al. 2011; Yoshino et al. 2017). Moreover, financial education, such as budgeting, risk assessment, and knowledge of the nature of random events, is important for gambling and, indeed, any financial decision (Hurla et al. 2017; Turner et al. 2008). Since gambling and financial decisions have similarities, and financial literacy and financial education have been proven to help people make informed financial decisions (Braunstein and Welch 2002; Lusardi et al. 2011; Yoshino et al. 2017), it is worth examining whether financial literacy and financial education could influence people’s gambling decisions. Therefore, financial literacy and financial education are our variables of interest in explaining gambling behavior.

To measure financial literacy, we use observed financial literacy following the methodology of Lusardi and Mitchell (2008, 2011), which emphasizes financial knowledge from an investment perspective. The three financial literacy questions used in this study measure respondents’ current level of financial knowledge, including (1) capacity to calculate interest rates, (2) understanding inflation, and (3) knowledge about risk diversification (Lusardi and Mitchell 2008, 2011). These questions are investment concepts that are somewhat different from savings behavior.

To measure financial education, we utilize a recall question that asks whether the respondents received financial education in elementary school. However, it should be noted that financial education in Japan is a government policy and has been present in schools since the 1950s (Messy and Monticone 2016). The objective of the Japanese school curriculum is to promote and teach students about savings using a children’s bank campaign (Messy and Monticone 2016). Students learn how to deposit and withdraw money, calculate interest, and understand the value of money. Hence, the children’s bank campaign is considered the first formal financial education in Japan.

## Data and Methodology

### Data

We used data from the 2010 Preference Parameters Study (PPS) of the Institute of Social and Economic Research at Osaka University. The PPS is an annual individual survey that collects information about socio-economic characteristics and preferences data, and the participants are representative of the Japanese population. In fact, the PPS is a panel survey, but the 2010 survey is the only one that asked questions about gambling behavior, financial literacy, and financial education in the same survey. Our unit of analysis is the individual, and our sample comprises 3687 individuals or approximately 68% of valid respondents in 2010 (N = 5386 individuals). We excluded 1699 individuals because of missing data for demographic and socio-economic variables.

#### Dependent Variable

The dependent variable in our study is gambling behavior. The PPS contained a question that asked “Do you gamble in lotteries or at casinos, or bet on sporting events or horse races?” and provided seven responses, where 1 means “do not gamble at all,” 2 means “used to gamble but not anymore,” 3 means “hardly gamble,” 4 means “gamble several times a year or so,” 5 means “gamble once a month or so,” 6 means “gamble once a week or so,” and 7 means “gamble almost every day.” To answer our research question, we grouped these responses into a binary scale using the low-risk limit proposed by Currie et al. (2006). In other words, respondents who answered 1–5 were coded as 0 or non-frequent gamblers, while those who answered 6 or 7 were coded as 1 or frequent gamblers. It should be noted that our measurement broadly defines gambling behavior following Currie et al. (2006) and the behavioral model of gambling developed by Hurla et al. (2017) due to the lack of data on amounts spent on gambling.

#### Independent Variables

There are two main variables of interest in our study: financial literacy and financial education. To measure financial literacy, we followed Lusardi and Mitchell’s (2008) methodology, which is simple and widely adopted in existing literature (e.g., Fornero and Monticone 2011; Lusardi and Mitchell 2011, 2014; Kadoya and Khan 2019; Kadoya et al. 2018, 2020; Klapper and Panos 2011; Sekita 2011), and used the following three questions.

- Suppose you had ¥10,000 in a savings account and the interest rate is 2% per year and you never withdraw money or receive interest payments. After 5 years, how much would you have in this account in total?
  More than ¥10,200 (correct answer)
  Exactly ¥10,200
  Less than ¥10,200
  Do not know
  Refuse to answer
- Imagine that the interest rate on your savings account was 1% per year and inflation was 2% per year. After 1 year, how much would you be able to buy with the money in this account?
  More than today
  Exactly the same
  Less than today (correct answer)
  Do not know
  Refuse to answer
- Please indicate whether the following statement is true or false. “Buying a company stock usually provides a safer return than buying a stock mutual fund.”
  True
  False (correct answer)
  Do not know
  Refuse to answer

The first two questions measure the respondent’s understanding of how compound interest works and the effect of inflation. Indeed, the questions help evaluate the understanding of economic concepts and basic numeracy (Lusardi and Mitchell 2008). The third question evaluates the respondent’s understanding of the concept of risk diversification. In this study, we assigned a score of 1 for each correct answer and 0 for each incorrect answer. We obtained the financial literacy variable by equally weighting the average scores of the three questions.

For financial education, the respondents were asked “Did you receive any compulsory financial education when you were in elementary school?” with three possible responses: yes, no, and do not know. We coded respondents who answered yes as 1 and respondents who answered no and do not know as 0, and treat this as a binary variable.

Furthermore, we included other control variables in our specifications: gender, age, university degree, marital status, household income, household assets, household members, and employment status. In addition, we controlled for the risky behaviors of smoking and drinking alcohol. The definitions of all variables are presented in Table 1.

**Table 1:** Variable definitions

Variables	Definitions
Gambling behavior	Binary variable: 1 = frequent gambler (gamble once a week or more) and 0 = otherwise
Financial literacy	Continuous variable: number of correct answers from three financial literacy questions
Financial education at school	Binary variable: 1 = yes and 0 = otherwise
Male	Binary variable: 1 = male and 0 = female
Age	Age of respondent
University degree	Binary variable: 1 = obtained university degree and 0 = otherwise
Marriage	Binary variable: 1 = marriage and 0 = otherwise
Divorce	Binary variable: 1 = divorced or separated and 0 = otherwise
Household income	Annual earned income before taxes and with bonuses of entire household in 2009 (unit: million JPY)
Household assets	Balance of financial assets (savings, stocks, insurance, etc.) of entire household (unit: million JPY)
Household members	Number of people currently living in household
Children	Binary variable: 1 = have child/children and 0 = otherwise
Unemployed	Binary variable: 1 = respondent is unemployed and 0 = otherwise
Smoking	Binary variable: 1 = smoke (sometimes–more than two packs a day) and 0 = do not smoke (do not smoke at all, quit, or hardly smoke)
Alcohol	Binary variable: 1 = drink (sometimes –five cans of beer everyday) and 0 = do not drink (do not drink at all or hardly drink)

### Descriptive Statistics

The sample descriptive statistics are reported in Table 2. Almost half the sample were men and on average, respondents were 49.82 years old. Approximately 27% of the sample had obtained a university degree. About 83% were currently married and only 3% had divorced. Respondents had approximately 6.5 million yen of annual household income on average and 13 million yen of household financial assets in 2009. They had an extended family, with 3.5 household members on average. More than 84% of the sample had children and only 2% were currently unemployed. On average, 24% of the sample smoked, while 54% drank alcohol.

**Table 2:** Descriptive statistics

Variables	Mean	SD	Min	Max
Gambling behavior	0.092	0.289	0	1
Financial literacy	0.592	0.344	0	1
Financial education at school	0.157	0.364	0	1
Male	0.491	0.500	0	1
Age	49.824	12.640	20	76
University degree	0.271	0.445	0	1
Marriage	0.825	0.380	0	1
Divorce	0.034	0.181	0	1
Household income	6.519	3.782	1	20
Household assets	13.181	17.609	2.5	100
Household members	3.523	1.439	1	10
Children	0.844	0.363	0	1
Unemployed	0.024	0.153	0	1
Smoking	0.241	0.428	0	1
Alcohol	0.539	0.499	0	1
**Observations**	**3687**			

Tables 3 and 4 report the distribution of gambling behavior classified by certain demographic characteristics and risky behaviors, respectively. In our sample, there were 340 frequent gamblers who gambled at least once a week (about 9% of the total sample) and 3347 non-frequent gamblers. There were significant differences in gambling behavior between men and women and among age groups. About 15% of male respondents were frequent gamblers compared to 4% of female respondents. More than 10% of respondents in the age groups of 41–50 and 51–60 years were frequent gamblers whereas in the other age groups, the proportion of frequent gamblers was less than 10%. Meanwhile, there were insignificant disparities in gambling behavior for respondents with different education levels. However, there were considerable differences in gambling behavior between smokers and non-smokers and between drinkers and non-drinkers. Specifically, more than 15% of smokers and more than 10% of drinkers were frequent gamblers. The differences in gambling behavior between those who were unemployed and those who were employed were insignificant.

**Table 3:** Distribution of gambling behavior classified by certain demographic characteristics

Gambling behavior	Gender		Education		Age					Total
	Female	Male	Lower than university degree	University degree and higher	≤ 30	31–40	41–50	51–60	≥ 61	
Non-frequent gambler	1803	1544	2434	913	252	630	822	849	794	3347
	(96.06%)	(85.30%)	(90.55%)	(91.39%)	(92.65%)	(92.11%)	(89.15%)	(89.27%)	(92.54%)	(90.78%)
Frequent gambler	74	266	254	86	20	54	100	102	64	340
	(3.94%)	(14.70%)	(9.45%)	(8.61%)	(7.35%)	(7.89%)	(10.85%)	(10.73%)	(7.46%)	(9.22%)
Total	1877	1810	2688	999	272	684	922	951	858	3687
	(100%)	(100%)	(100%)	(100%)	(100%)	(100%)	(100%)	(100%)	(100%)	(100%)
Mean difference	t-value = −11.4791***		t-value = 0.7841		F-value = 2.81**					

Note: *** p < 0.01, ** p < 0.05, * p < 0.10

This table reports the numbers and percentages of respondents classified by demographic features: gender, education, and age

**Table 4:** Distribution of gambling behavior classified by risky behaviors

Gambling behavior	Smoking		Alcohol		Unemployed		Total
	No	Yes	No	Yes	No	Yes	
Non-frequent gambler	2598	749	1574	1773	3265	82	3347
	(92.89%)	(84.16%)	(92.70%)	(89.14%)	(90.72%)	(93.18%)	(90.78%)
Frequent gambler	199	141	124	216	334	6	340
	(7.11%)	(15.84%)	(7.30%)	(10.86%)	(9.28%)	(6.82%)	(9.22%)
Total	2797	890	1698	1989	3599	88	3687
	(100%)	(100%)	(100%)	(100%)	(100%)	(100%)	(100%)
Mean difference	t-value = −7.9023***		t-value = −3.7268***		t-value = 0.7886		

Note: *** p < 0.01, ** p < 0.05, * p < 0.10

This table reports the numbers and percentages of respondents classified by risky behaviors: smoking, drinking alcohol, and being unemployed

## Methods

Our research seeks to investigate how financial literacy and financial education are related to gambling behavior. First, we separately estimate the effects of financial literacy and financial education in Eqs. (1) and (2), respectively. We then include both financial literacy and financial education to see the combined effect of the variables in Eq. (3).

1 $$Y_{i} = f\left( {FL_{i} ,X_{i} ,\varepsilon_{i} } \right)$$

2 $$Y_{i} = f\left( {FE_{i} ,X_{i} ,\varepsilon_{i} } \right)$$

3 $$Y_{i} = f\left( {FL_{i} ,FE_{i} ,X_{i} ,\varepsilon_{i} } \right)$$

where Y is the gambling behavior of the i respondent (frequent or non-frequent gamblers); FL represents the score on the financial questions measuring financial literacy; FE represents financial education at school; X is a vector of individual characteristics; and $\varepsilon$ is the error term. Since the dependent variable is a binary choice, we employed a probit model to estimate all equations.

However, it can be argued that including financial literacy as an explanatory variable in Eqs. (1) and (3) could create an endogeneity bias owing to an undefined causal relationship between financial literacy and gambling behavior. Hurla et al. (2017) argued that both gambling and financial decisions are related to risk assessment and that most people have difficulty accurately evaluating risks and probabilities to maximize expected payoffs in both situations. As a result, individuals with lower financial literacy tend to become frequent gamblers more easily owing to low risk assessment skills. On the other hand, individuals who are frequent gamblers might acquire financial savvy through their gambling experiences. To overcome this possible issue, we applied the instrumental variable (IV) method to our specifications using the education of the respondent’s father as an IV. Studies by Lusardi et al. (2010) and Mahdavi and Horton (2014) found that financial literary is significantly correlated with parental education. Based on our sample, the regression of financial literacy on father’s education together with other control variables shows that father’s education is statistically significant at the 5% level. However, the regression of gambling behavior on father’s education and other control variables shows that there is no significant impact of father’s education on gambling behavior. The results have not been reported in the paper to save space but are available upon request. Since it has been proven that father’s education is related to a respondent’s financial literacy but not to gambling behavior, father’s education is used as an IV in this study. Thus, in this case, we employed a probit-IV to estimate Eqs. (1) and (3).

Our full model specifications are

1a $$\begin{aligned} & Gambling\:behavior_{i} \left( {1 = frequent\:gambler\:and\:0 = non - frequent\:gambler} \right) \\ & \quad = \beta_{0} + \beta_{1} financial\:literacy_{i} \left( {IV = father^{\prime}s\:education} \right) + \beta_{2} male_{i} + \beta_{3} age_{i} \\ & \quad \quad + \beta_{4} university\:degree_{i} + \beta_{5} marriage_{i} + \beta_{6} divorce_{i} + \beta_{7} household\:income_{i} \\ & \quad \quad + \beta_{8} household\:assets_{i} + \beta_{9} household\:members_{i} + \beta_{10} children_{i} + \beta_{11} unemployed_{i} \\ & \quad \quad + \beta_{12} smoking_{i} + \beta_{13} alcohol_{i} + \beta_{14} smoking*alcohol_{i} + \varepsilon_{i} \\ \end{aligned}$$

2a $$\begin{aligned} & Gambling\:behavior_{i} \left( {1 = frequent\:gambler\:and\:0 = non - frequent\:gambler} \right) \\ & \quad = \beta_{0} + \beta_{1} financial\:education_{i} + \beta_{2} male_{i} + \beta_{3} age_{i} + \beta_{4} university\:degree_{i} \\ & \quad \quad + \beta_{5} marriage_{i} + \beta_{6} divorce_{i} + \beta_{7} household\:income_{i} + \beta_{8} household\:assets_{i} \\ & \quad \quad + \beta_{9} household\:members_{i} + \beta_{10} children_{i} + \beta_{11} unemployed_{i} + \beta_{12} smoking_{i} \\ & \quad \quad + \beta_{13} alcohol_{i} + \beta_{14} smoking*alcohol_{i} + \varepsilon_{i} \\ \end{aligned}$$

3a $$\begin{aligned} & Gambling\:behavior_{i} \left( {1 = frequent\:gambler\:and\:0 = non - frequent\:gambler} \right) \\ & \quad = \beta_{0} + \beta_{1} financial\:literacy_{i} \left( {IV = father^{\prime}s\:education} \right) + \beta_{2} financial\:education_{i} \\ & \quad \quad + \beta_{3} male_{i} + \beta_{4} age_{i} + \beta_{5} university\:degree_{i} + \beta_{6} marriage_{i} + \beta_{7} divorce_{i} \\ & \quad \quad + \beta_{8} household\:income_{i} + \beta_{9} household\:assets_{i} + \beta_{10} household\:members_{i} \\ & \quad \quad + \beta_{11} children_{i} + \beta_{12} unemployed_{i} + \beta_{13} smoking_{i} + \beta_{14} alcohol_{i} + \beta_{15} smoking*alcohol_{i} + \varepsilon_{i} \\ \end{aligned}$$

## Empirical Results

In this section, we report the individual effects of financial literacy and financial education in Tables 5 and 6, respectively, and combine the effects of both financial literacy and financial education in Table 7. Each table presents the results of three different specifications of the explanatory variables.

**Table 5:** Regression results of gambling behavior when financial literacy is the main explanatory variable

VARIABLES	PROBIT			IVPROBIT (father’s education as IV)		
	Model 1.1	Model 1.2	Model 1.3	Model 1.4	Model 1.5	Model 1.6
Financial literacy	− 0.00893	− 0.00904	− 0.000607	− 2.316***	− 2.308***	− 2.230**
	(0.0911)	(0.0911)	(0.0913)	(0.822)	(0.826)	(0.953)
Male	0.752***	0.752***	0.668***	0.688***	0.690***	0.664***
	(0.0676)	(0.0676)	(0.0755)	(0.163)	(0.162)	(0.137)
Age	0.000634	0.000640	0.00173	0.00804**	0.00806**	0.00837**
	(0.00292)	(0.00292)	(0.00296)	(0.00350)	(0.00353)	(0.00353)
University degree	− 0.237***	− 0.237***	− 0.222***	0.151	0.150	0.134
	(0.0746)	(0.0746)	(0.0750)	(0.190)	(0.190)	(0.203)
Marriage	0.0672	0.0743	0.0669	0.137	0.186*	0.173
	(0.112)	(0.134)	(0.132)	(0.0860)	(0.106)	(0.109)
Divorce		0.0232	− 0.0312		0.153	0.127
		(0.220)	(0.219)		(0.169)	(0.183)
Household income	0.0167*	0.0167*	0.0177**	0.0455***	0.0455***	0.0447***
	(0.00891)	(0.00892)	(0.00902)	(0.0104)	(0.0105)	(0.0113)
Household assets	− 0.00994***	− 0.00994***	− 0.00959***	− 0.00308	− 0.00308	− 0.00324
	(0.00254)	(0.00254)	(0.00249)	(0.00454)	(0.00455)	(0.00479)
Household members	− 0.0105	− 0.0103	− 0.00861	− 0.0575**	− 0.0562**	− 0.0537**
	(0.0239)	(0.0240)	(0.0238)	(0.0242)	(0.0242)	(0.0265)
Children	− 0.0820	− 0.0870	− 0.105	− 0.158*	− 0.194*	− 0.209**
	(0.119)	(0.132)	(0.131)	(0.0923)	(0.104)	(0.106)
Unemployed			− 0.184			− 0.294
			(0.228)			(0.179)
Smoking			0.478***			0.282
			(0.111)			(0.192)
Alcohol			0.0915			0.0826
			(0.0813)			(0.0669)
Smoking * Alcohol			− 0.329**			− 0.276**
			(0.135)			(0.138)
Constant	− 1.693***	− 1.696***	− 1.830***	− 0.408	− 0.441	− 0.567
	(0.179)	(0.182)	(0.186)	(0.763)	(0.757)	(0.884)
**Observations**	3,687	3,687	3,687	3,687	3,687	3,687
**Log likelihood**	− 1051	− 1051	− 1040	− 2076	− 2075	− 2058
**Chi2 statistics**	154.9	156.1	182.8	518.7	516.3	519.9
**p-value**	0	0	0	0	0	0
**Exogeneity test**				2.65	2.64	2.07
**p-value**				0.1035	0.1044	0.1499
**AIC**	2121.053	2123.041	2109.258	4196.189	4197.151	4179.081
**BIC**	2183.178	2191.379	2202.447	4332.866	4346.253	4377.884

Note: Robust standard errors are reported in parentheses; *** p < 0.01, ** p < 0.05, * p < 0.10

**Table 6:** Regression results of gambling behavior when financial education is the main explanatory variable

VARIABLES	PROBIT		
	Model 2.1	Model 2.2	Model 2.3
Financial education at school	− 0.189**	− 0.189**	− 0.181**
	(0.0902)	(0.0902)	(0.0910)
Male	0.751***	0.751***	0.669***
	(0.0675)	(0.0675)	(0.0752)
Age	0.00140	0.00140	0.00246
	(0.00292)	(0.00292)	(0.00297)
University degree	− 0.238***	− 0.237***	− 0.221***
	(0.0742)	(0.0741)	(0.0747)
Marriage	0.0753	0.0816	0.0749
	(0.112)	(0.134)	(0.132)
Divorce		0.0210	− 0.0326
		(0.220)	(0.219)
Household income	0.0167*	0.0167*	0.0179**
	(0.00877)	(0.00878)	(0.00887)
Household assets	− 0.0100***	− 0.0100***	− 0.00965***
	(0.00253)	(0.00253)	(0.00248)
Household members	− 0.00827	− 0.00808	− 0.00671
	(0.0239)	(0.0241)	(0.0239)
Children	− 0.0840	− 0.0885	− 0.107
	(0.118)	(0.131)	(0.131)
Unemployed			− 0.173
			(0.228)
Smoking			0.476***
			(0.111)
Alcohol			0.0886
			(0.0814)
Smoking * Alcohol			− 0.330**
			(0.135)
Constant	− 1.721***	− 1.724***	− 1.853***
	(0.177)	(0.181)	(0.184)
**Observations**	3,687	3,687	3,687
**Log likelihood**	− 1048	− 1048	− 1038
**Chi2 statistics**	157	158.1	183.8
**p-value**	0	0	0
**AIC**	2116.51	2118.5	2105.141
**BIC**	2178.636	2186.839	2198.329

Note: Robust standard errors are reported in parentheses; *** p < 0.01, ** p < 0.05, * p < 0.10

**Table 7:** Regression results of gambling behavior when both financial literacy and financial education are the main explanatory variables

VARIABLES	PROBIT			IVPROBIT (father’s education as IV)		
	Model 3.1	Model 3.2	Model 3.3	Model 3.4	Model 3.5	Model 3.6
Financial literacy	− 0.00570	− 0.00580	0.00191	− 2.344***	− 2.336***	− 2.265**
	(0.0910)	(0.0910)	(0.0912)	(0.779)	(0.783)	(0.898)
Financial education at school	− 0.189**	− 0.189**	− 0.181**	− 0.0977	− 0.0981	− 0.0987
	(0.0901)	(0.0901)	(0.0909)	(0.0931)	(0.0932)	(0.0960)
Male	0.751***	0.752***	0.669***	0.682***	0.685***	0.660***
	(0.0677)	(0.0677)	(0.0755)	(0.161)	(0.159)	(0.137)
Age	0.00141	0.00142	0.00245	0.00854***	0.00857***	0.00887***
	(0.00293)	(0.00293)	(0.00298)	(0.00322)	(0.00324)	(0.00324)
University degree	− 0.237***	− 0.237***	− 0.221***	0.158	0.157	0.142
	(0.0748)	(0.0748)	(0.0753)	(0.182)	(0.183)	(0.194)
Marriage	0.0755	0.0819	0.0748	0.142*	0.191*	0.179*
	(0.112)	(0.134)	(0.132)	(0.0846)	(0.104)	(0.106)
Divorce		0.0211	− 0.0327		0.154	0.129
		(0.220)	(0.219)		(0.168)	(0.180)
Household income	0.0168*	0.0168*	0.0179**	0.0458***	0.0459***	0.0451***
	(0.00891)	(0.00892)	(0.00901)	(0.00993)	(0.0100)	(0.0107)
Household assets	− 0.0100***	− 0.0100***	− 0.00965***	− 0.00298	− 0.00298	− 0.00311
	(0.00254)	(0.00254)	(0.00250)	(0.00442)	(0.00443)	(0.00465)
Household members	− 0.00841	− 0.00822	− 0.00667	− 0.0568**	− 0.0555**	− 0.0532**
	(0.0239)	(0.0240)	(0.0239)	(0.0240)	(0.0240)	(0.0261)
Children	− 0.0842	− 0.0887	− 0.107	− 0.161*	− 0.197*	− 0.212**
	(0.118)	(0.131)	(0.131)	(0.0910)	(0.102)	(0.104)
Unemployed			− 0.173			− 0.287
			(0.228)			(0.178)
Smoking			0.476***			0.274
			(0.111)			(0.187)
Alcohol			0.0886			0.0804
			(0.0814)			(0.0659)
Smoking * Alcohol			− 0.330**			− 0.274**
			(0.135)			(0.137)
Constant	− 1.719***	− 1.722***	− 1.854***	− 0.395	− 0.428	− 0.546
	(0.179)	(0.182)	(0.186)	(0.745)	(0.739)	(0.860)
**Observations**	3,687	3,687	3,687	3,687	3,687	3,687
**Log likelihood**	− 1048	− 1048	− 1038	− 2073	− 2072	− 2055
**Chi2 statistics**	157.2	158.3	183.9	552.4	549	550.3
**p-value**	0	0	0	0	0	0
**Exogeneity test**				2.9	2.89	2.3
**p-value**				0.0885	0.0892	0.1293
**AIC**	2118.507	2120.497	2107.14	4194.7	4195.649	4178.049
**BIC**	2186.845	2195.048	2206.542	4343.801	4357.176	4389.276

Note: Robust standard errors are reported in parentheses; *** p < 0.01, ** p < 0.05, * p < 0.10

### Financial Literacy

In the first three columns of Table 5, we report the results of the probit regressions using financial literacy as the main explanatory variable. Overall, there are no differences in the significance of the estimated parameters among the three specifications. The coefficients of our variable of interest, financial literacy, are negative but insignificant. To deal with possible endogeneity bias, as discussed in the previous section, we present the results of the probit-IV regressions in the last three columns of Table 5, which use father’s education as an IV for financial literacy. Overall, there are considerable differences in the results of the probit and probit-IV regressions. The coefficients of financial literacy in the probit-IV regressions are negative and strongly significant. Of the other variables, male, age, and household income have a considerably positive relationship with frequent gambling, while household members and children have a significantly negative relationship in all specifications.

In column 5 of Table 5, divorce was added as an independent variable because some researchers have claimed that marital dissolution may cause people to engage in harmful activities (e.g., Castren et al. 2017). The regression results are similar to those in column 4; although divorce is insignificant, marriage is weakly significant. In column 6, we include additional independent variables, such as unemployment, smoking, and drinking alcohol, since previous studies have suggested that job insecurity and risky behaviors could explain gambling behavior (e.g., Castren et al. 2017; Clarke et al. 2006; Feigelman et al. 1995). The results in column 6 show that smoking, drinking alcohol, and unemployment are insignificant but the interaction term between smoking and alcohol has a significantly negative relationship with frequent gambling.

The results obtained from the probit-IV model differ considerably from those of the probit model, implying that there is endogeneity bias in Eq. (1). To check the robustness of the results, we examine whether our instrument is valid. By controlling for demographic and socio-economic factors, we find that father’s education is significantly related to financial literacy but not to gambling behavior in OLS and probit regressions, respectively. Hence, father’s education is a valid IV.

### Financial Education

Table 6 shows the results of the probit regression when financial education is used as the main explanatory variable. In this case, there is no endogeneity problem because financial education in school occurred in the past and is used to explain current gambling behavior. Conversely, current gambling behavior cannot affect financial education that occurred when respondents were young. Hence, we can identify the causal relationship between financial education and gambling behavior in this case, and there is no evidence of endogeneity bias. Therefore, the IV is not necessary in this model.

Overall, there are no differences in the significance of the estimated parameters among the three specifications. The coefficients of our variable of interest, financial education, are negative and statistically significant at the 5% level. Male and household income still have significantly positive impacts while the interaction term between smoking and alcohol has a significantly negative impact on frequent gambling, which is similar to Models 1.4–1.6 in Table 5. However, age, household members, and children become insignificant while university degree, household assets, and smoking become significant.

The results in Tables 5 and 6 show that both financial literacy and financial education have a significant impact on gambling behavior. To explore the relationship between these two variables, we regress financial literacy on financial education and other control variables. We find that financial literacy and financial education are not correlated, consistent with Sekita (2011). Therefore, both financial literacy and financial education should be used in the same equation to explain gambling behavior, as we show in our final model in the next subsection.

### Financial Literacy and Financial Education

Table 7 shows the results of probit and probit-IV regressions when both financial literacy and financial education are used as the main explanatory variables. In the first three columns of Table 7, the results from the probit regressions show that the coefficients of financial literacy and financial education are negative but only the coefficients of financial education are significant, which is similar to the results of the probit regressions in Tables 5 and 6, respectively.

However, after resolving the endogeneity bias by using father’s education as an IV, the results differ considerably. In the last three columns of Table 7, the results from the probit-IV regressions show that the coefficients of financial literacy and financial education are negative but only the coefficients of financial literacy are significant. For other variables, the results are similar to the probit-IV regression results in Table 5. The coefficients of male, age, marriage, and household income have positive relationships, while household members and children have significantly negative relationships with frequent gambling. For health risk behavior variables, it is worth noting that the individual effect of smoking and alcohol (smoking = 1 and alcohol = 0; and smoking = 0 and alcohol = 1) has a positive but insignificant impact on frequent gambling. However, co-consumption, that is, smoking and drinking alcohol together (smoking = 1 and alcohol = 1), represented by the interaction term between smoking and alcohol, has a significantly negative impact on frequent gambling.

In addition to endogeneity issues, we also examine multicollinearity among the independent variables. There may be a linear association between the inclusion of financial literacy and economic outcomes (Kadoya et al. 2018). For example, individuals with high education levels could have high financial savvy, or those with high net worth may have more financial knowledge because of experience with asset management. Moreover, the inclusion of smoking and alcohol consumption could have a collinear relationship (Grucza and Bierut 2006). In other words, individuals who smoke may also drink alcohol. However, we found no multicollinearity, as the variance inflation factors (VIF) of the variables (not reported) are far below 10.

## Discussion

There is an important difference between financial education and the observed level of financial literacy in this study in the context of Japan. Both variables have a significant impact on our estimations, and therefore, we include both financial literacy and financial education as explanatory variables in Table 7. If we compare the coefficients of the probit-IV regressions in Tables 5 and 7, they all have similar signs, levels of significance, and magnitudes. However, the results of the probit-IV regressions in Table 7 show that when both financial literacy and financial education are included as explanatory variables, the coefficients of financial education become insignificant while the coefficients of financial literacy remain significant. This implies that gambling behavior is better explained by financial literacy than by financial education.

The results from the probit-IV estimations in Models 3.4–3.6 show that financial literacy has a significantly negative impact on gambling behavior. In other words, if an individual has a high level of financial literacy, he or she has a lower probability of becoming a frequent gambler. Our results suggest that financial literacy with a focus on an investment perspective is more effective in mitigating gambling behavior compared to financial education that focuses on savings.

Moreover, household members, children, and the interaction term between smoking and alcohol have a significantly negative relationship with gambling behavior. On the other hand, male, age, marriage, and household income have a significantly positive relationship with gambling behavior. University degree, divorce, household assets, unemployment status, smoking, and alcohol consumption have no significant relationship with gambling behavior.

Our results on gender are consistent with existing studies that found a higher gambling disorder rate among males (see, e.g., APA 2013; Castren et al. 2017; Currie et al. 2006; Toneatto and Nguyen 2007). Some empirical studies support the association between people with lower socioeconomic status and low income and higher rates of problem gambling (e.g., Currie et al. 2006; Toneatto and Nguyen 2007). However, our results are contradictory in the case of household income (positive coefficient). One reason for this could be that people with high incomes have a high demand for leisure (Houston and Wilson 2002), and gambling is considered a form of leisure activity.

A significant relationship has been found in existing studies between health risk behaviors and problem gambling (see, e.g., Castren et al. 2017; Thompson et al. 2005) and in our results in the case of co-consumption, that is, smoking and drinking alcohol together (negative coefficient). Nonetheless, the impacts of health risk behavior on gambling are still inconclusive. Interpreting health risk behavior coefficients is difficult because of the undefined causal relationship between health risk behavior and gambling behavior. Moreover, they may have a confounding relationship with other variables that may be related to problem gambling; this should be further explored.

## Conclusion

This study examines the relationship between financial literacy, financial education, and gambling behavior among the Japanese population. We hypothesize that financially literate and financially educated people who use their knowledge to make sound financial decisions are less likely to gamble. However, the results suggest that financial literacy can explain gambling behavior better than financial education. The results from the probit-IV model show that financial literacy has a significantly negative relationship with gambling frequency. In other words, a high level of financial literacy (with emphasis on knowledge of investments) significantly reduces gambling frequency. However, receiving financial education in elementary school (with an emphasis on savings behavior) has no significant relationship with gambling behavior in the Japanese context.

Our findings suggest that problem gambling could be mitigated by promoting financial literacy, but no such conclusion could be drawn for financial education. It should be noted that there is an important difference between financial education and financial literacy in Japan. This claim is supported by Sekita’s (2011) empirical research on the relationship between financial literacy and retirement planning in Japan. Sekita (2011) showed that receiving financial education through a children’s bank campaign has no effect on the level of financial literacy among representative Japanese adults. However, financial education is positively correlated with having savings plans for retirement and the results hold even after considering the level of financial literacy (Sekita 2011). As a result, financial education helps people save but financial literacy is more effective in reducing gambling. Therefore, the government might integrate investment knowledge into the current financial education program to empower the impact of financial literacy. In addition, further research is needed to explore the effect of financial literacy on different groups of gamblers. Since gambling has non-financial motives as well (Neighbors et al. 2002; Nower and Blaszczynski 2010), it is worth investigating gambling motivations to examine whether financial literacy has different impacts on gamblers who have different motives (e.g., financial vs. non-financial motives) and different levels of gambling severity. This research would help to prevent problem gambling in the future.

This study has limitations. First, we used only three questions to measure the level of financial literacy. However, these three questions, designed by Lusardi and Mitchell (2008, 2011), have been used to measure financial literacy in many countries and have made financial literacy comparable internationally (e.g., Almenberg and Säve-Söderbergh 2011; Crossan et al. 2011; Fornero and Monticone 2011; Kadoya and Khan 2019; Kadoya et al. 2018; Klapper and Panos 2011; Sekita 2011; Lusardi and Mitchell 2014). Second, we used only one question on the frequency of gambling to measure gambling behavior. This is because the PPS is the only survey in Japan that contained questions on gambling behavior, financial education, financial literacy, and demographic variables. Nevertheless, based on data availability, our study is a pioneer work that provides supporting evidence for the government’s promotion of financial literacy.

## References

[CR1] Alessi S, Petry N (2003). Pathological gambling severity is associated with impulsivity in a delay discounting procedure. Behavioural Processes.

[CR2] Almenberg J, Säve-Söderbergh J (2011). Financial literacy and retirement planning in Sweden. Journal of Pension Economics & Finance.

[CR3] American Psychiatric Association (2013). Diagnostic and statistical manual of mental disorders (DSM-5).

[CR4] Asahi Shimbun. (2018). Casino bill must address concerns about gambling addiction. Retrieved 27 November 2018, from http://www.asahi.com/ajw/articles/AJ201805220029.html.

[CR5] Braunstein S, Welch C (2002). Financial literacy: An overview of practice, research, and policy. Federal Reserve Bulletin.

[CR6] Calado F, Griffiths MD (2016). Problem gambling worldwide: An update and systematic review of empirical research (2000–2015). Journal of Behavioral Addictions.

[CR7] Cassetta BD, Kim HS, Hodgins DC, McGrath DS, Tomfohr-Madsen LM, Tavares H (2018). Disordered gambling and psychosis: Prevalence and clinical correlates. Schizophrenia Research.

[CR8] Castren S, Kontto J, Alho H, Salonen AH (2017). The relationship between gambling expenditure, socio-demographics, health-related correlates and gambling behavior—A cross-sectional population-based survey in Finland. Addiction.

[CR9] Churchill SA, Farrell L (2018). The impact of gambling on depression: New evidence from England and Scotland. Economic Modelling.

[CR10] Clarke D, Tse S, Abbott M, Townsend S, Kingi P, Manaia W (2006). Key indicators of the transition from social to problem gambling. International Journal of Mental Health and Addiction.

[CR11] Crossan D, Feslier D, Hurnard R (2011). Financial literacy and retirement planning in New Zealand. Journal of Pension Economics and Finance.

[CR12] Cunningham-Williams RM, Cottler LB, ComptonIII WM, Spitznagel EL (1998). Taking chances: Problem gamblers and mental health disorders-results from the St Louis Epidemiologic Catchment Area Study. American Journal of Public Health.

[CR13] Currie SR, Hodgins DC, Wang J, El-Guebaly N, Wynne H, Chen S (2006). Risk of harm among gamblers in the general population as a function of level of participation in gambling activities. Addiction.

[CR14] Eber GB, Shaffer HJ (2000). Trends in bio-behavioral gambling studies research: Quantifying citations. Journal of Gambling Studies.

[CR15] Economist. (2017). The world’s biggest gamblers. Retrieved 20 December 2018, from http://www.economist.com/graphic-detail/2017/02/09/the-worlds-biggest-gambler.

[CR16] Feigelman W, Kleinman PH, Lesieur HR, Millman RB, Lesser ML (1995). Pathological gambling among methadone patients. Drug and Alcohol Dependence.

[CR17] Fornero E, Monticone C (2011). Financial literacy and pension plan participation in Italy. Journal of Pension Economics and Finance.

[CR18] Frederick S, Loewenstein G, O’Donoghue T (2002). Time discounting and time preference: A critical review. Journal of Economic Literature.

[CR19] Grucza RA, Bierut LJ (2006). Cigarette smoking and the risk for alcohol use disorders among adolescent drinkers. Alcoholism, Clinical and Experimental Research.

[CR20] Haydock M, Cowlishaw S, Harvey C, Castle D (2015). Prevalence and correlates of problem gambling in people with psychotic disorders. Comprehensive Psychiatry.

[CR21] Herrnstein RJ, Prelec D, Loewenstein G, Elster J (1992). A theory of addiction. Choice over time.

[CR22] Houston RG, Wilson DP (2002). Income, leisure and proficiency: An economic study of football performance. Applied Economics Letters.

[CR23] Hurla, R., Kim, M., Singer, E., & Soman, D. (2017). Applying findings from financial literacy to encourage responsible gambling. In *Research report series behavioural economics in action* (pp. 367–380). Rotman School of Management, University of Toronto.

[CR24] Imai A (2018). Casinos in Japan. *Lancet*. Psychiatry.

[CR25] Kadoya Y, Khan MSR (2018). Can financial literacy reduce anxiety about life in old age?. Journal of Risk Research.

[CR26] Kadoya Y, Khan MS (2019). What determines financial literacy in Japan?. Journal of Pension Economics and Finance.

[CR27] Kadoya Y, Khan MSR, Hamada T, Dominquez A (2018). Financial literacy and anxiety about life in old age: Evidence from the USA. Review of Economics of the Household.

[CR28] Kadoya Y, Khan MSR, Oba H, Narumoto J (2020). Factors affecting knowledge about the adult guardianship and civil trust systems: evidence from Japan. Journal of Women and Aging.

[CR29] Kessler RC, Hwang I, LaBrie R, Petukhova M, Sampson NA, Winters KC, Shaffer HJ (2008). DSM-IV pathological gambling in the National Comorbidity Survey Replication. Psychological Medicine.

[CR30] Klapper L, Panos GA (2011). Financial literacy and retirement planning: the Russian case. Journal of Pension Economics and Finance.

[CR31] Leeman RF, Potenza MN (2012). Similarities and differences between pathological gambling and substance use disorders: A focus on impulsivity and compulsivity. Psychopharmacology (Berl).

[CR32] Lusardi, A., Michaud, P. C., & Mitchell, O. (2011). Optimal financial literacy and saving for retirement. RAND Working Paper Series, No. WR-905-SSA.

[CR33] Lusardi A, Mitchell OS (2008). Planning and financial literacy: How do women fare?. American Economic Review.

[CR34] Lusardi A, Mitchell OS (2011). Financial literacy around the world: An overview. Journal of Pension Economics and Finance.

[CR35] Lusardi A, Mitchell OS (2014). The economic importance of financial literacy: Theory and evidence. Journal of Economic Literature.

[CR36] Lusardi A, Mitchell OS, Curto V (2010). Financial literacy among the young. Journal of Consumer Affairs.

[CR37] Mahdavi M, Horton NJ (2014). Financial knowledge among educated women: Room for improvement. Journal of Consumer Affairs.

[CR38] Manzenreiter W (2013). Playing against all odds: Pachinko and the culture of risk-taking in Japan’s crisis economy. Leisure Studies.

[CR39] Martin RJ, Usdan S, Nelson S, Umstattd MR, LaPlante D, Perko M, Shaffer H (2010). Using the theory of planned behavior to predict gambling behavior. Psychology of Addictive Behaviors.

[CR40] Massachusetts Council on Compulsive Gambling (MCCG). (n.d.). Financial literacy. https://masscompulsivegambling.org/services/financial-literacy/. Retrieved from 27 November 2018.

[CR41] Messy, F. A., & Monticone, C. (2016). Financial education policies in Asia and the Pacific. OECD Working Papers on Finance, Insurance and Private Pensions, 40. Paris: OECD Publishing.

[CR42] Moodie C, Finnigan F (2006). Association of pathological gambling with depression in Scotland. Psychological Reports.

[CR43] Neighbors C, Lostutter TW, Cronce JM, Larimer ME (2002). Exploring college student gambling motivation. Journal of Gambling Studies.

[CR44] North American Foundation for Gambling Addiction Help (NAFGAH). (n.d.) Gambling and gambling addiction. http://nafgah.org. Retrieved from 27 November 2018.

[CR45] Nower L, Blaszczynski A (2010). Gambling motivations, money-limiting strategies, and precommitment preferences of problem versus non-problem gamblers. Journal of Gambling Studies.

[CR46] Osaki, T. (2018). Casinos in Japan: Tourist attractions or hotbeds of gambling addiction? http://www.japantimes.co.jp/news/2018/07/16/national/casinos-japan-tourist-attractions-hotbeds-gambling-addiction/#.XBfgkS-B3y8. Accessed 27 November 2018.

[CR47] Sekita S (2011). Financial literacy and retirement planning in Japan. Journal of Pension Economics & Finance.

[CR48] Shaffer HJ (2005). From disabling to enabling the public interest: Natural transitions from gambling exposure to adaptation and self-regulation. Addiction.

[CR49] Shaffer HJ, Stanton MV, Nelson SE (2006). Trends in gambling studies research: Quantifying, categorizing, and describing citations. Journal of Gambling Studies.

[CR50] Tanaka, N. (2018). Gambling addiction in the land of Pachinko. https://www.nippon.com/en/currents/d00367/. Accessed 27 November 2018.

[CR51] Thaler R (1981). Some empirical evidence on dynamic inconsistency. Economics Letters.

[CR52] Thompson WN, Tanioka I, Fujimoto K (2005). Pachinko players in Japan: Subculture, cult, or ordinary citizens at leisure?. Gaming Law Review.

[CR53] Toneatto T, Nguyen L, Smith G, Hodgins DC, Williams RJ (2007). Individual characteristics and problem gambling behavior. Research and measurement issues in gambling studies.

[CR54] Turner NE, Macdonald J, Somerset M (2008). Life skills, mathematical reasoning and critical thinking: A curriculum for the prevention of problem gambling. Journal of Gambling Studies.

[CR55] Yoshino, N., Morgan, P. J., & Trinh, L. Q. (2017). Financial literacy in Japan: Determinants and impacts. ADBI Working Paper Series, 796, Tokyo: Asian Development Bank Institute.

